# The Value of Radio Frequency Identification in Quality Management of the Blood Transfusion Chain in an Academic Hospital Setting

**DOI:** 10.2196/medinform.9510

**Published:** 2019-08-05

**Authors:** Linda W Dusseljee-Peute, Remko Van der Togt, Bas Jansen, Monique W Jaspers

**Affiliations:** 1 Academic Medical Center- Amsterdam Department of Medical Informatics University of Amsterdam Amsterdam Netherlands

**Keywords:** radio waves, automatic data processing, blood transfusion, geographic information systems, temperature, technology, guideline adherence

## Abstract

**Background:**

A complex process like the blood transfusion chain could benefit from modern technologies such as radio frequency identification (RFID). RFID could, for example, play an important role in generating logistic and temperature data of blood products, which are important in assessing the quality of the logistic process of blood transfusions and the product itself.

**Objective:**

This study aimed to evaluate whether location, time stamp, and temperature data generated in real time by an active RFID system containing temperature sensors attached to red blood cell (RBC) products can be used to assess the compliance of the management of RBCs to 4 intrahospital European and Dutch guidelines prescribing logistic and temperature constraints in an academic hospital setting.

**Methods:**

An RFID infrastructure supported the tracking and tracing of 243 tagged RBCs in a clinical setting inside the hospital at the blood transfusion laboratory, the operating room complex, and the intensive care unit within the Academic Medical Center, a large academic hospital in Amsterdam, the Netherlands. The compliance of the management of 182 out of the 243 tagged RBCs could be assessed on their adherence to the following guidelines on intrahospital storage, transport, and distribution: (1) RBCs must be preserved within an environment with a temperature between 2°C and 6°C; (2) RBCs have to be transfused within 1 hour after they have left a validated cooling system; (3) RBCs that have reached a temperature above 10°C must not be restored or must be transfused within 24 hours or else be destroyed; (4) unused RBCs are to be returned to the BTL within 24 hours after they left the transfusion laboratory.

**Results:**

In total, 4 blood products (4/182 compliant; 2.2%) complied to all applicable guidelines. Moreover, 15 blood products (15/182 not compliant to 1 out of several guidelines; 8.2%) were not compliant to one of the guidelines of either 2 or 3 relevant guidelines. Finally, 148 blood products (148/182 not compliant to 2 guidelines; 81.3%) were not compliant to 2 out of the 3 relevant guidelines.

**Conclusions:**

The results point out the possibilities of using RFID technology to assess the quality of the blood transfusion chain itself inside a hospital setting in reference to intrahospital guidelines concerning the storage, transport, and distribution conditions of RBCs. This study shows the potentials of RFID in identifying potential bottlenecks in hospital organizations’ processes by use of objective data, which are to be tackled in process redesign efforts. The effect of these efforts can subsequently be evaluated by the use of RFID again. As such, RFID can play a significant role in optimization of the quality of the blood transfusion chain.

## Introduction

Blood transfusion is a common, even lifesaving, approach for the treatment of patients with severe conditions and in need of blood from others. Despite the development of modern technology significantly facilitating the process of blood transfusion, risks such as the mismatch of blood type and disqualification of red blood cell (RBC) products quality still exist. In Europe, Directive 2002/98/EC of the European Commission states that member states shall take all necessary measures to ensure that blood and blood components in hospital settings comply to the requirements on the storage, transport, and distribution conditions of blood and blood components [1]. As an example, guideline 2004/33/EC from the European Commission prescribes that RBCs to be used for transfusion should be stored at temperatures between 2°C and 6°C [2]. In addition, transport and distribution of blood and blood components at all stages of the transfusion chain must be under conditions that maintain the quality of the product [2].

These guidelines concerning the storage, transport, and distribution conditions of RBCs are needed to prevent bacterial growth in these products as much as possible. Bacterial growth in RBCs that are stored at 4ºC is slow and limited to a few gram-negative organisms that rapidly proliferate at cold temperatures [3]. There are reports on the effect of RBC storage up to 25ºC for varying periods of time [4]. These studies show that RBCs stored for 24 hours at 25ºC reduces the shelf life of RBCs by 1 week [5,6]. Although there is no evidence indicating the relevance of keeping RBCs’ temperature below 10ºC for quality assurance, it has been argued to be unlikely that short-term exposures of RBCs to 10ºC will have a negative impact on their quality [4]. Another study by Hamill showed that there might be a lag phase of up to 4 hours before bacteria begin to grow exponentially in RBCs, after being spiked with various bacteria and moved from an environment of 1ºC-6ºC to an environment of 24ºC [7]. Overall, these studies show that there is a relation between the storage temperature of RBCs and the risk of bacterial growth when moved into environments with higher temperatures for a specific time period. To reduce bacterial growth and the risk of transfusion-related sepsis, the storage of RBCs at low temperatures should be standard practice [8].

In the Netherlands, Sanquin is responsible for blood supply on a not-for-profit basis and advances transfusion medicine as such that it fulfills the highest demands for quality, safety, and efficiency [9]. RBCs produced by Sanquin are to adhere to European Directives 2002/98/EC and 2004/33/EC as mentioned above [10]. Therefore, Sanquin has set the following guidelines concerning the storage, transport, and distribution conditions of RBCs inside the transfusion chain. First, RBCs must be preserved within an environment with a temperature between 2°C and 6°C to prevent bacterial growth [10]. Second, RBCs that have reached a temperature above 10°C must not be restored or must be transfused within 24 hours or else be destroyed. Third, during transport, the temperature conditions of RBCs must be remained, unless they are transfused immediately after cross-matching activities have been performed. Fourth, intrahospital blood banks should not keep RBCs outside cooling systems longer than half an hour [11]. Therefore, hospitals are allowed to place cooling systems at wards or operating theatres [11].

Table 1 gives an overview of these European and Dutch guidelines concerning storage, transport, and distribution conditions of RBCs.

A quality process called “hemovigilance” ensures that the entire transfusion chain is continually controlled and that safety procedures and treatment practices are updated [12-15]. Although guidelines are in place, bottlenecks concerning intrahospital storage, transport, and distribution of RBCs remain, resulting in outdating of RBCs. Sandler argues that although requirements of written directives concerning blood collections and transfusions are followed for decades, this does not guarantee that RBCs are managed as required: “the surge of technologic complexity in all steps in the blood collection-transfusion loop has created a situation in which simply following what the book says” does not ensure that the blood will be collected or transfused as required [16]. Current blood transfusion management information systems are poor in supporting traceability of RBCs as a result of missing transfusion and distribution forms, variation in the availability and validity of transfusion information, and ambiguous information concerning the location of RBC transfusion [17]. These facts emphasize that a significant improvement of RBCs’ traceability could come from a better compliance to the rules of information transmission. Other studies have noted that current safeguards to prevent mistransfusions are inadequate [18-22].

A significant improvement in blood transfusion safety could come from automated systems supplying the relevant information to assess the adherence to guidelines, including those prescribing logistic and temperature constraints concerning the blood transfusion loop [14,16,18-22].

The most common form of automated identification, labeling, and processing RBCs is barcode technology, which has demonstrated a reduction in blood management and supply chain problems and mistransfusion errors [18]. Unfortunately, broader adoption of this technology has been hindered due to its limitations [18,20]. Barcodes on medical products, for instance, have the disadvantage that they require active user interaction and that they must be read in a straight line (line-of-sight) [14,20,21]. Moreover, multiple barcodes on products, including those codes containing irrelevant information from previous steps in the process, might generate incorrect information when the wrong barcode is being scanned further in the process [22].

**Table 1:** Overview of national and international guidelines concerning intrahospital storage, transport, and distribution conditions for red blood cell products.

Source [Reference]	Organization	Guideline(s)
Directive 2002/98/EC of the European parliament and of the council [1]	European Union	Article 22: Blood establishments shall ensure that the storage, transport and distribution conditions of blood and blood components comply with the requirements referred to in Article 29(e)
		Article 29: The following technical requirements and their adaption to technical and scientific progress shall be decided in accordance with the procedure referred to in Article 28(2): ... (e) storage, transport and distribution requirements.
Commission directive 2004/33/EC [2]	European Union	Article 5: Blood establishments shall ensure that the storage, transport and distribution conditions for blood and blood components comply with the requirements set out in Annex 4.
		Annex 4—1.1 Liquid storage: Temperature of storage concerning red cells preparations and whole blood (if used for transfusion as whole blood): + 2 to + 6°C; 2. Transport and Distribution: Transport and distribution of blood and blood components at all stages of the transfusion chain must be under conditions that maintain the integrity of the product.
Blood guide Part 1 erythrocytes, platelets, fresh frozen plasma [10]	Sanquin, Dutch national blood bank	Red blood cell products must be preserved within an environment with a temperature between 2°C and 6°C to prevent bacterial growth.
		Red blood cell products that have reached a temperature above 10°C must not be restored or must be transfused within 24 hours or else be destroyed.
		During transport, the temperature conditions of red blood cell products must be remained, unless they are transfused immediately after cross-matching activities have been performed.

Recommendations from literature for enhancing the safety of blood transfusions include further research evaluating the merits of technological innovations based on, for instance, radio frequency identification (RFID) [14-17,19,22-29]. Compared with information systems requiring manual data entry, RFID is a more advanced and effective technique in RBC management than a barcode system. First, RFID tags can hold more and more up-to-date information and can generate more accurate data [30]. In addition, information about objects tagged with, for example, RFID can be transmitted for multiple objects simultaneously, through physical barriers and from a distance, something which is impossible to realize with barcodes [28].

RFID further enables automated monitoring of the location and storage temperature of blood products within the distribution chain of a facility or during transportation [18,28]. Finally, the implementation of RFID in the blood transfusion chain could, for instance, reduce the number of incorrect blood components transfused by using smart pumps that read RFID-coded data placed on blood bags and a patient’s wristband [22].

A complex process like the blood transfusion chain could benefit from modern technologies such as RFID. RFID could play an important role in generating logistic and temperature data of randomized controlled trials (RCTs), which are important in assessing the quality of the logistic process of blood transfusions and the product itself. Until recently, in the Academic Medical Center (AMC) of Amsterdam, logistic data on RCTs was collected on paper and temperature data of RCTs was not collected at all. Within this study, we examined the merits of RFID technology in generating data required for assessing the quality of the blood transfusion chain in the AMC, that is, logistic and temperature data on RCTs. We expect that data generated by tracing RFID-tagged blood products enable us to monitor the intrahospital process of the transfusion chain. The assessment of the quality of the RBC product itself, which depends on its storing conditions through the blood transfusion chain, can be realized by collecting temperature data. Overall, these datasets can be used for assessing the quality of the blood transfusion chain by verifying its compliance to current guidelines and regulations. To our knowledge, thus far, studies assessing the compliance of the blood transfusion chain to guidelines using location and temperature data collected by use of RFID technology within this chain have not been performed.

The overall aim of this study was to evaluate the merits of RFID for the generation of data used in assessing the compliance to guidelines concerning the quality indicators of the management of RBCs inside a hospital environment. More specifically, the aim was to evaluate whether location, time stamp, and temperature data generated in real time by an active RFID system containing temperature sensors attached to RBCs could be used to assess the compliance of the management of RBCs to 4 intrahospital guidelines prescribing logistic and temperature constraints in the AMC.

## Methods

### Background

This study was performed within the AMC, a large academic hospital in Amsterdam, the Netherlands. The AMC is a 1002 beds hospital including 21 outpatient clinics, 34 inpatient clinics, 5 day care units, employing 960 full-time equivalent clinicians in total. The study was part of the project “RFID in health care.” This project was initiated and sponsored by the Dutch Ministry of Health, Welfare and Sport. This study was part of this project and gathered data at the blood transfusion laboratory (BTL), the operating room complex, and the intensive care unit (ICU) of the AMC.

### Intrahospital Guidelines

In our study, we assessed the compliance of RBCs to the following guidelines concerning intrahospital storage, transport, and distribution of RBCs, which were derived from [10,11]: (1) RBCs must be preserved within an environment with a temperature between 2°C and 6°C; (2) RBCs have to be transfused within 1 hour after they have left a validated cooling system; (3) RBCs that have reached a temperature above 10°C must not be restored or must be transfused within 24 hours or else be destroyed; (4) unused RBCs are to be returned to the blood transfusion laboratory within 24 hours after they left the transfusion laboratory.

### Active Radio Frequency Identification System

RFID systems exist of 3 main parts: (1) the tag, which is the identification device attached to the object being tracked; (2) the reader that recognizes the nearby presence of a tag and reads and processes the data that are stored on the tag; and (3) the antenna, which is part of the communication between the tag and the reader [31]. The RFID system implemented in our setting was manufactured as an active system that uses RFID tags with temperature sensors containing batteries to communicate their identity, location, and temperature information to nearby readers using radio waves. The electromagnetic field covers a distance ranging from 1 m to 30 m. The RFID system (Eureka RFID, Avonwood, England) had a 125-kHz reader (68 x 10E-3 μT at 1 m) that forces tags to transmit in its proximity. The active RFID tag had an operational frequency of 868 MHz at 2 μW. For a more extensive description of the RFID infrastructure, we refer to the study by Marjamaa et al [30]. After data transmission by a tag to a receiver, data including its battery status are sent through the local area network to a database. Every 8 min, the tag’s temperature sensor would additionally record temperature data in its memory for final storage in this database. To spare the tag’s battery life, the tag is activated to start recording and deactivated to stop recording at the beginning and the end of the process.

The selection of the RFID system was based on the following. First, the RFID tag must contain a temperature sensor covering the possible temperature ranges of RBCs. Second, the data concerning location, temperature, and time stamp should be generated by the tag without the need of intervention by a person, that is, who had to scan the tag data actively. The intervention of a person is required when tags are only able to broadcast their data over short ranges, that is, passive tags. Third, its operation is needed to fulfill the requirements of the overall project including contemporary integration with the local communications network of the AMC and provision of accurate data concerning a product’s location within the health care facilities BTL, 3 ICUs, and 2 operating rooms.

### Tracking and Tracing of Red Blood Cells With Radio Frequency Identification

**Figure 1:**
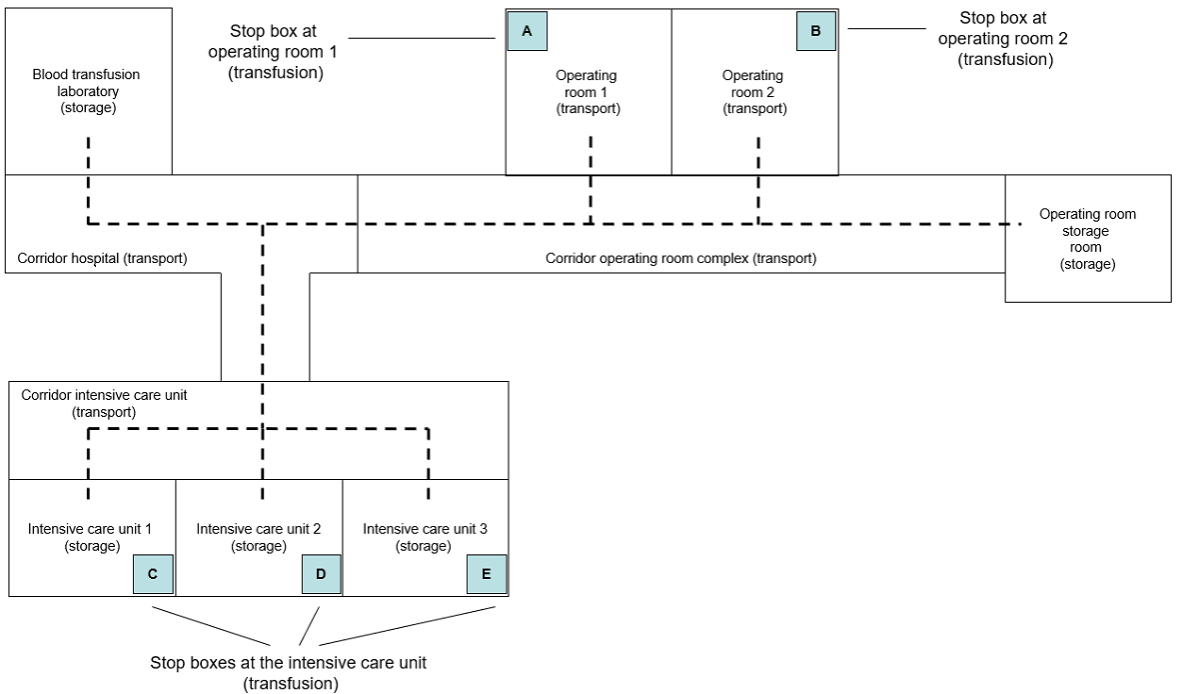
Schematic floor plan and tracking routes of RBCs tagged with active RFID tags withtemperature sensors at the Blood Transfusion laboratory, Operating Rooms and Intensive Care Units.

The RFID infrastructure supported the tracking and tracing of 243 tagged RBCs in a clinical setting at the BTL, the operating room complex, and the ICU at the AMC. These tagged RBCs were tracked from the moment they left the BTL (hospital blood bank) until they were transfused into a patient at the operating room or ICU or were returned to the BTL for reuse. The blood products were transported between different storage rooms and between storage rooms and rooms where the transfusion took place. RBCs were stored inside official refrigerators available in the storage rooms. The tags were activated in the BTL when being attached to a specific blood bag. In the pilot study, tags were activated by laboratory assistants. After activation, the tags started recording temperatures every 8 min. The laboratory assistant verified the activation of the tag on a computer screen. Before an RBC was transfused, the tag was separated from the blood product and put into a so-called “stop box” by the hospital staff member held responsible for the transfusion of the RBC to the intended patient. When an RBC was returned to the BTL for reuse, the tag was separated from the blood product and deactivated by the laboratory assistant. The tag stopped generating data as soon as it was put into the so-called stop box or had been deactivated. A schematic floor plan with the different potential tracks followed by tagged RBCs is depicted in Figure 1.

### Collection and Storage of Radio Frequency Identification–Generated Data

Data concerning real-time location, time stamp, and temperature were generated by tags allocated to blood products and stored into an Oracle database every time these tags would pass a RFID reader. The dataset generated was split into subsets of data containing blood product storage data, transport data, or transfusion data. Blood storage data concerned the data on tagged blood products located inside a room where an official validated refrigerator was available. It was assumed that when blood products were inside storage rooms, they were placed inside the refrigerator. Storage data concerned all data generated inside the storage rooms at the operating room and the ICU, but also the data generated inside the BTL itself. Transport data concerned all data on tagged RBCs during their transportation between storage rooms. Transfusion data concerned all data generated by tagged blood products between the time they had left a storage room until the time they were transfused.

### Radio Frequency Identification Data Quality Assessment

For identification and locating blood products and temperature measurements, an indoor RFID system was used. The RFID system should discriminate between blood products located in different rooms inside the hospital building and track the transition of blood products between these rooms in the right sequence. Therefore, at each side of a door of passage, a reader was placed to generate a “gate way.” The specification of the required accuracy concerning (blood product) location data was inferred from the different activities that take place inside different hospital rooms, that is, transport at corridors, storage inside storage rooms, and transfusions inside the operating room and ICU. The specification of the required accuracy of the corresponding time stamps was to realize the generation of data about the whereabouts and conditions of blood products as close to reality as possible.

A study on the quality of data concerning time, location, and temperature generated by the RFID technology implemented in our hospital setting preceded the pilot study [32].

First, we assessed whether our RFID system generated accurate data for the tracking and tracing of RBCs inside the AMC. The first set of tests on the accuracy of temperature data generated by the RFID tags revealed that real-time recorded temperature data were on average 0.26ºC and 0.5ºC higher compared with the temperature data measured by an official data logger and a quicksilver thermometer, respectively. The second set of tests showed that the RFID tags adjusted with an average speed of −0.42°C per minute when moved from an environment of 23°C to an environment of 5°C. The results of the tests indicated that the active tags were able to monitor the whole range of temperatures that blood products could achieve in the transfusion chain. Furthermore, based on the accuracy test concerning the speed of temperature adjustment, it was assumed that the tags were able to adjust to new temperature circumstances more quickly than the blood products themselves [32]. In general, both sets of tests showed that the data generated by the active tags were accurate enough for monitoring the RBCs’ temperatures and whereabouts inside our hospital setting.

Second, we assessed all datasets that were generated by the RFID tags on their completeness within the real-life clinical setting, namely, the blood transfusion chain in the AMC. Overall, the completeness of the RFID generated datasets concerning location, time, and temperature of the blood products varied from 90% to 100%; datasets from only 13 tags were missing after they had left a certain location within the facility [32].

Third, the previous study, likewise, showed that our RFID technology was capable of generating accurate location, temperature, and time data [32]. To guarantee valid statements concerning the compliance of the blood transfusion chain to safety guidelines, in this study, incomplete datasets were excluded.

On the basis of this previous research, within this study, the following criteria concerning RBC temperature data generated by the tags were taken into account: (1) data on collected temperatures were included when the tag had been allocated to the RBC product longer than 1 hour; (2) temperatures generated by RFID within the range of 1.5°C to 6.5°C were considered as being compliant to temperature constraints as prescribed by guideline 1; (3) temperature data from transfusion datasets were excluded from our study because RBCs concentrates are warmed up just before being transfused, resulting in temperatures higher than 6°C; and (4) temperatures generated by RFID with a value of 11°C were considered as being noncompliant to temperature constraints as prescribed by guideline 3. Considering the aims of our study, location and time stamp data did not need any adjustments or cleaning because the previous tests concerning the accuracy of location and time data generated by active RFID tags had shown minimum differences compared with hand-recorded time and location data.

### Dataset Selection and Cleanness

The datasets generated by tagged RBCs were analyzed for their suitability concerning the assessment of the compliancy of the management of RBCs within the AMC with the 4 European and Dutch guidelines on logistic and temperature constraints. The inclusion of the datasets generated by the RFID system was based on the following conditions: (1) tagged RBCs had produced data after they had left the BTL until they were transfused or returned unused to the BTL; (2) the datasets generated by the tagged RBCs could be split up in either storage room data, transport data, or transfusion data; and (3) the tagged RBCs produced complete datasets for assessment of the quality of the blood transfusion chain in the context of guideline 1.

All datasets generated by the tagged blood products that did not meet conditions 1, 2, or 3 were excluded from the analysis.

This resulted in an inclusion of datasets generated by tracking 182 RBCs (182/243 blood product datasets included; 74.9%). The reasons for excluding the datasets generated by the other 61 blood products were the following: (1) 7 tagged RBCs did not leave the BTL; (2) 13 tagged RBCs were “lost” after they left the BTL; (3) the datasets concerning 40 tagged RBCs that were finally transfused at the ICU could not be split up in storage and transfusion data subsets, as the stop box was placed inside the storage room at the ICU; and (4) all sub datasets generated by 1 tagged blood product were incomplete.

The remaining 182 tagged blood products generated 416 datasets. In addition, 18 of these datasets, which were generated by 15 RBCs (18/416 incomplete datasets; 4.3%), were incomplete and were excluded from the analysis.

### Guideline Compliancy Assessment With Radio Frequency Identification Data

To assess the compliance of the AMC blood transfusion chain to guideline 1, the datasets of a total of 182 blood products, transfused or returned to the BTL, were analyzed. For these 182 complete datasets (182/182 complete data; 100%) containing transport and storage data, the amount of RBCs that had maintained a temperature between the range of 1.5°C and 6.5°C was calculated.

To assess the compliance of the blood transfusion chain to guidelines 2 and 3, the datasets of 52 tagged blood products finally transfused at the operating room (52/182 transfused; 28.6%) were analyzed. Of these 52 datasets containing transfusion data, 50 were complete (96%), for which the amount of RBCs that had spent less than 1 hour outside storage rooms with official cooling systems until the moment they had been transfused was calculated. Of these 52 datasets containing storage, transport, and transfusion data, 48 were complete (92%), for which the amount of RBCs that had been transfused within 24 hours after they had exceeded a temperature of 11°C was calculated.

A total of 130 blood products finally returned to the BTL unused (130/182 returned to lab; 71.4%). These datasets were used to assess to what extent the blood transfusion chain in the AMC complies with guideline 4. Of these 130 datasets containing storage and transport data, 118 were complete (118/130 complete data; 90.8%), for which the amount of RBC products that had not been transfused and were returned to the BTL within 24 hours was calculated.

### Data Analyses

**Figure 2:**
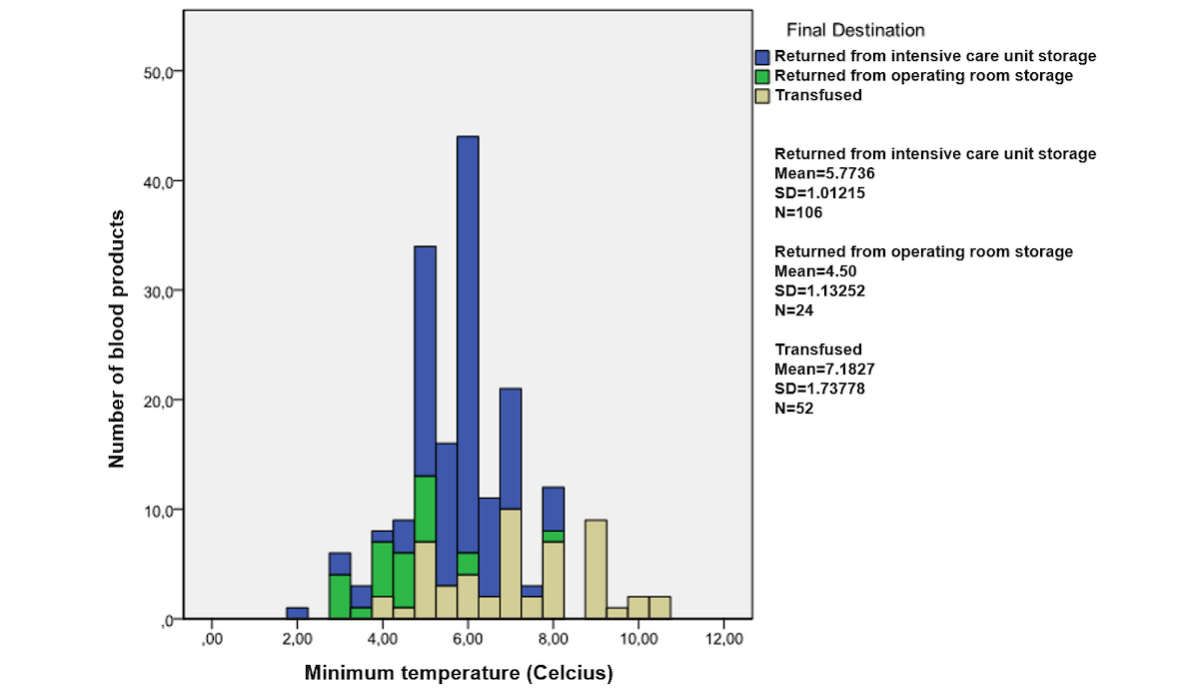
Guideline 1; distribution of the minimum temperatures measured by radio frequency identification (RFID) tags attached to red blood cells.

**Figure 3:**
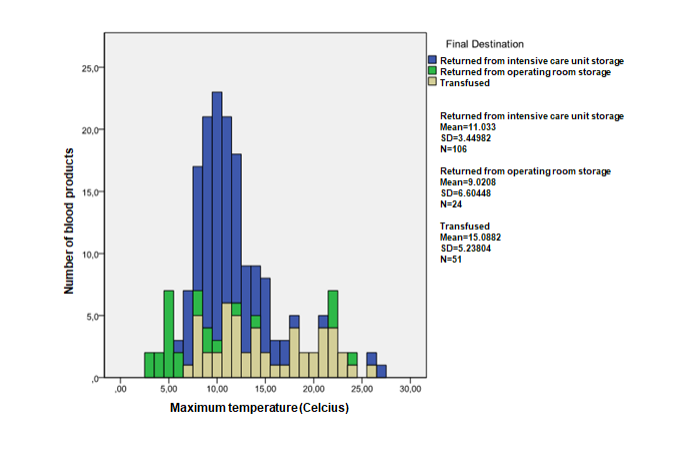
Guidelines 1 and 3; distribution of the maximum temperatures measured by radio frequency identification (RFID) tags attached to red blood cells.

**Figure 4:**
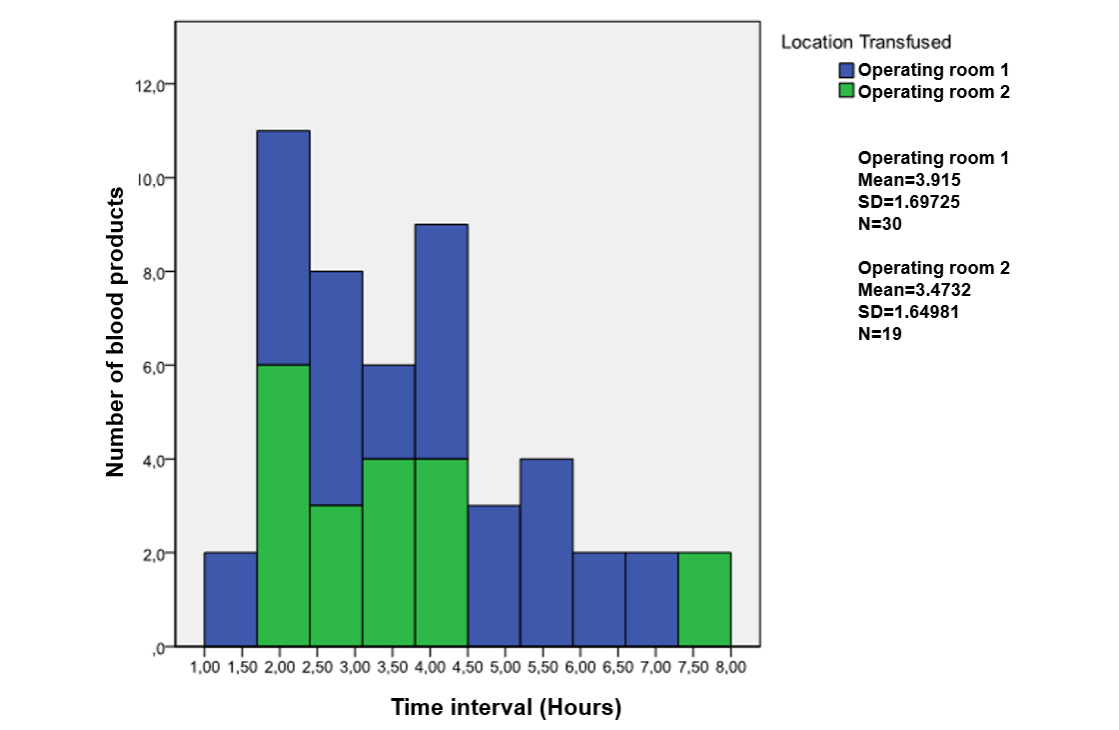
Guideline 2; distribution of the time intervals generated by radio frequency identification (RFID) tags until the red blood cell had been transfused in the operating room.

**Figure 5:**
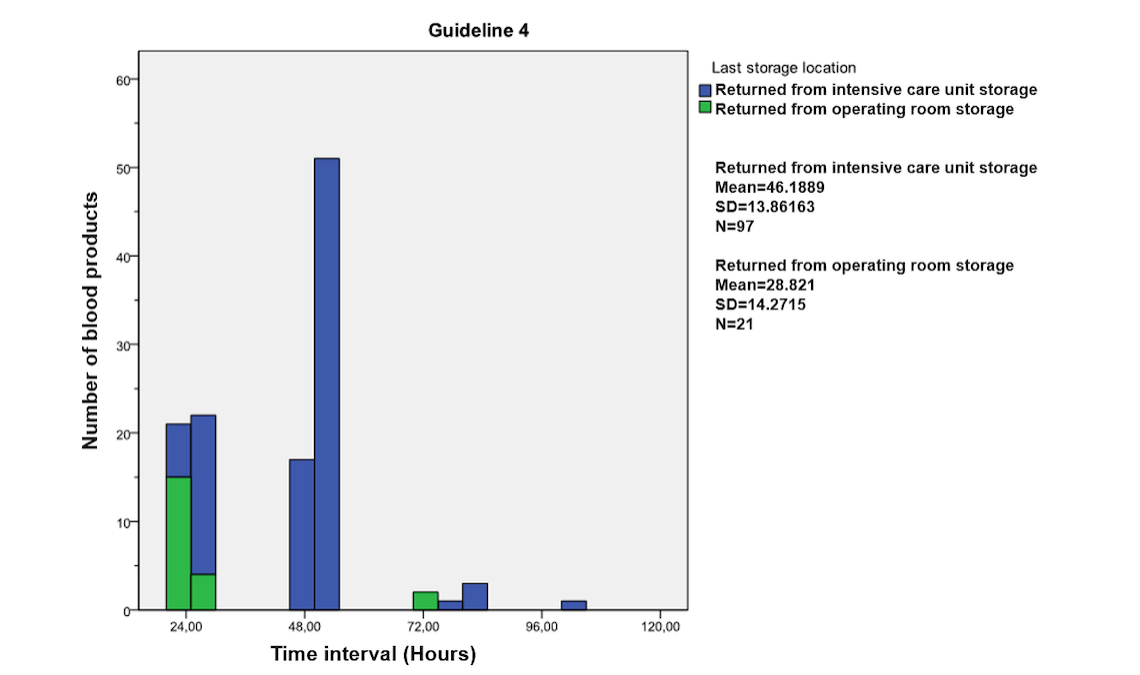
Guideline 4; distribution of the time intervals generated by radio frequency identification (RFID) until the red blood cell returned from the operating room or intensive care unit to the Blood Transfusion Laboratory.

On the basis of the analyses of the datasets generated by RFID, the RBCs were split into 2 groups, those that complied and those that did not comply with the specified guidelines. A decision tree was used to classify the RBCs concerning their compliancy with all relevant guidelines. For each guideline and for different subgroups, mean, maximum, and minimum values were registered, which are displayed in Table 2 and graphs displayed in Figures 2-5.

**Table 2:** A schematic overview of the number of red blood cells that were managed in relation to their applicable guidelines (N=182).

Applicable guideline(s)	Managed red blood cells, n (%)
Compliant to all applicable guidelines	4 (2.2)
Compliant to 1 out of 2 or 2 out of 3 applicable guidelines	15 (8.2)
Compliant to none out of 2 or 1 out of 3 applicable guidelines	148 (81.3)
Datasets left out of the analysis	15 (8.2)

## Results

### Overview

In total, the management of 4 blood products (4/182 compliant; 2.2%) complied to all applicable guidelines. These 4 blood products returned unused to the transfusion laboratory and their management fully complied to guidelines 1 and 4. The management of 15 blood products (15/182 not compliant to 1 out of several guidelines; 8.2%) was not compliant to 1 of the guidelines of either 2 or 3 relevant guidelines, 5 were compliant to guideline 4 but not to guideline 1 and 10 to guideline 1 but not to guideline 4. Finally, the management of 148 blood products (148/182 not compliant to 2 guidelines; 81.3%) was not compliant to 2 relevant guidelines, 99 were not compliant to guidelines 1 and 4 and 49 were not compliant to guidelines 1 and 2 but were compliant to guideline 3. In total, 15 tagged blood products (15/182 missing data; 8.2%) produced incomplete datasets, and their management could, therefore, not be used to assess their compliance to each of the applicable guidelines. Table 2 provides an overview of the number of RBCs that were managed according to the applicable guidelines.

The following paragraphs will elaborate on the compliancy of the management of RBCs with regard to each individual guideline. A schematic overview of the compliancy of blood products to each of these guidelines is depicted in Multimedia Appendix 1.

### Guideline 1

RBCs must be preserved within an environment with a temperature between 2°C and 6°C [10]. Data generated by 182 blood products (182 complete of 182 datasets; 100%) were used to assess the compliance of the management of the blood transfusion chain to guideline 1. The management of 16 (16/182 datasets; 8.8%) blood products (minimum temperature range 3.0°C-6.0°C; maximum temperature range 3.0°C-6.5°C) complied to guideline 1. The management of the other 166 blood products (166 noncompliant of 182 datasets; 91.2%) did not comply to this guideline (minimum temperature range 2.0°C-10.5°C; maximum temperature range 7.0°C-27.0°C).

An overview of the number of blood products and their minimum and maximum temperature distributions is depicted in Figures 2 and 3, respectively. First, these graphs show that both minimum and maximum temperatures are distributed normally concerning the management of all RBCs. Second, mean temperatures (mean minimum temperature 7.2°C, mean maximum temperature 15.1°C) are highest for RBCs that were finally transfused, followed by the temperatures of the RBCs that returned from the ICU (mean minimum temperature 5.8°C, mean maximum temperature 11.1°C) and operating room (mean minimum temperature 4.5°C, mean maximum temperature 9.0°C).

### Guideline 2

Guideline 2 states that RBCs have to be transfused within 1 hour after they have left a validated cooling system (AMC guideline). Data generated by 49 blood (49/52 complete data; 94%) were used to assess the compliance of the blood transfusion chain to guideline 2. None of the 49 blood products (100%) did comply to this guideline (mean 3.74 hours; range 1.11-7.56).

Figure 4 provides an overview of the number of blood products in relation to the different time intervals that these products spent outside a validated cooling system before they were transfused at an operating room. It shows that the difference in mean temperature between RBCs until transfusion at operating room 1 (mean 3.9°C) and operating room 2 (mean 3.5°C) is 0.4°C.

### Guideline 3

Guideline 3 states that RBCs that have reached a temperature above 10°C must not be restored or must be transfused within 24 hours or else be destroyed [10].

In total, 100 RBCs (100/182 in total; 55.0%) exceeded a temperature of 10.0°C. The datasets of 49 blood products (49/52 complete data; 94%) were used to assess the compliance of the blood transfusion chain to guideline 3 concerning their transfusions. A total of 39 out of these 49 blood products (39/49; 80%) had exceeded a temperature of 10.0°C (range 7.0-26.0°C). All 49 blood products were yet transfused within 24 hours, resulting in a 100% compliance of RBCs to the maximum temperature and maximum time allowed for transfusion after having left the BTL of the hospital.

Figure 3 shows the distribution concerning the maximum temperatures measured by the RFID tag attached to the RBC product. It shows that maximum temperatures concerning RBCs that were transfused varied from 7°C to 26°C.

### Guideline 4

Guideline 4 states that unused RBCs are to be returned to the BTL within 24 hours after they left the transfusion laboratory (AMC guideline). The datasets of 118 blood products (118 complete of 130 datasets; 90.8%) were analyzed to assess the compliance of the blood transfusion chain to guideline 4, resulting in 9 compliant (9/118 compliant; 7.6%) blood products (mean 23.42 hours, range 23.12-23.83). The other 109 blood products (109/118 noncompliant; 92.4%) did not comply to this guideline (mean 44.72 hours, range 24.03-102.91).

Figure 5 provides an overview of the number of blood products in relation to the different time intervals concerning that these RBCs returned from an operating room or ICU to the BTL. It shows that the difference in mean time between RBCs that return from the operating room (mean 28.8 hours) and ICU (mean 46.2 hours) is 17.4 hours.

## Discussion

### Principal Findings

This study evaluated the merits of RFID in assessing the compliance to 4 intrahospital guidelines based on current European and national Dutch guidelines concerning the management of RBCs inside an academic hospital setting. RBCs were tagged with active RFID tags with temperature sensors and generated location, time stamp, and temperature data in real time at the operating room and ICU of the AMC.

The management of only 2% of all assessed RBCs complied to all applicable guidelines. In all, 8.2% of all assessed RBCs were not compliant to 1 guideline of either 2 or 3 relevant guidelines, whereas 81.3% of the RBCs were not compliant to 2 applicable guidelines. Overall, this study revealed that an information system based on active RFID capable of generating location, temperature, and time stamp data by tracking RBCs in real time can be used for assessing the management of RBCs to guidelines concerning the quality of the blood transfusion chain.

In the AMC, a previous lack of detailed information required to monitor and evaluate recommended minimum and maximum temperature levels, time periods spent outside official cooling systems, and recommended time lapses before transfusion is the main reason that the management of a majority of the RBCs did not comply to the guidelines. Specific bottlenecks in, the previous mainly paper-based blood transfusion management information system mainly concerned incompletely filled-in or lost paper-based feedback forms. This led to variations in the availability and validity of information. As a result, the quality of the blood transfusion chain in the AMC concerning time and temperature constraints could not be guaranteed and specific process bottlenecks causing incompliancy to these guidelines not be identified.

On the basis of the data generated by the RFID system, several bottlenecks were revealed, more specifically, a considerably higher amount of RBCs that returned from the ICU to the BTL exceeded a temperature of 10°C as compared with RBCs that returned from the operating rooms to the BTL. Second, the RFID data showed that none of the RBCs were transfused within 1 hour after they had left a validated cooling system. Third, more unused RBCs that returned from the ICU to the BTL exceeded the time limit of 24 hours as compared with unused RBCs that returned from the operating rooms to the BTL. Focus groups with a team of stakeholders (BTL, operating room, and ICU units) are planned to examine and discuss these process bottlenecks more thoroughly and their underlying causes. On the basis of the discussion of the causes underlying the incompliancy of the management of RBCs to certain guidelines, possible process redesign efforts of the blood transfusion chain will be proposed and undertaken. After redesign of the blood transfusion chain, the RFID system and methods presented in this study will then allow follow-up assessments of the effects of these efforts on the quality of the blood transfusion chain in the AMC.

Other studies have successfully implemented RFID technology in blood transfusion medicine [16,18,22,33,34] but did not focus on revealing the merits of RFID in assessment of the compliancy to blood chain quality guidelines, including those prescribing logistic and temperature constraints. Sandler showed that radiofrequency microchips can collect key data concerning, for example, the donor, the manufacturing, laboratory test results, and expiration data during blood collections; can facilitate information transfer between blood centers and hospitals; and confirm recipient blood unit match at the bed side [16]. Dzik indeed proved that RFID technology can be used for prevention of bedside transfusion errors [22]. Briggs further showed that using RFID inside the blood transfusion chain can increase productivity, quality, and patient safety through RFID-enabled processes. The use of RFID technology reduced morbidity and mortality effects substantially among patients receiving transfusions [33]. In addition, Davis et al concluded that RFID will gain more productivity through more efficient processes and avoidance of time-consuming error and recovery and follow-up. Quality gains are due to avoidance of products’ discards caused by process errors and better inventory management [18]. Davis did yet not explicitly focus on the compliance of RFIDs to guidelines. Hohberger demonstrated a typical blood transfusion model based on RFID technology, covering the whole blood transfusion process from donation sites to hospital transfusion sites [34].

### Strengths

The strength of the RFID system implemented in this study is its versatility. In contrast to other studies, we showed the merits of using time stamp, temperature, and location data generated by RFID to assess compliance to quality guidelines concerning RBCs management. RFID systems as presented in this study could also be applied to assess the efficiency or quality of other hospital processes. With the real-time data these systems can produce, hospitals and other health care organizations can track and identify assets and patients, saving time of hospital staff in searching for equipment and monitoring patients. As such, RFID systems similar to the one used in this study might be helpful in optimizing clinical work flows, achieving operational efficiency, and improving patient safety. When RFID data would be linked to clinical or economic data in available hospital databases, more comprehensive evaluations of specific hospital processes could be realized, as well as areas of inefficiency, high cost or identification of potential patient safety compromising situations and redesign of (clinical and operational) workflows.

### Limitations

A limitation of this study concerns data quality issues. Due to missing data, about 8% of the datasets generated by the RFID tags could not be included in the analysis. In our previous study, we showed that our RFID system was capable of generating accurate and complete time stamp, location, and temperature data in a controlled laboratory and simulated field setting, results of which we could not reproduce in the real-life setting due to uncontrollable conditions [32]. One of the main reasons that led to the exclusion of these datasets in the field study had to do with the use of the “stop box” that was placed in each storage room at the ICU and operating rooms. First, the tags of 3 blood products were dropped in the stop box only after 4 to 6 days after the transfusion of the blood products had taken place at 1 of the operating rooms. Their datasets were, therefore, left out of the analysis. Certain other RFID datasets might yet have been incorrect due to these uncontrollable variations in the field setting, potentially resulting in an overestimation of violations of guidelines 2 and 3. First, conservative tuning of readers’ signals forcing tags to transmit their data can prevent signal overlap with other nearby readers and possible harmful electromagnetic interference on medical equipment. At the same time, this might result in weak signal coverage, which might have induced poor activation of tags, causing loss of transmission of identification and real-time location, time, and temperature data.

Moreover, the signals of tags broadcasting identification and real-time location, time, and temperature data might have been blocked by the blood product itself or other local circumstances like, for instance, by a person carrying the blood bag or several blood bags at once. Such blocked signals might not have reached the RFID receiver, and this might have resulted in a loss of blood bag identification and real-time location, time stamp, and temperature data. As can be inferred from the data and discussions with hospital staff, in practice, hospital staff put and kept the tag in their pocket a certain time period before dropping it in the stop box and even at another stop box than the one located in the room where the blood transfusion actually had taken place. In these specific cases, the RFID tags could not “tell us” that it was detached from the RBC. This issue will be taken into account in the process redesign effort of the blood transfusion chain in the AMC.

Second, 12 datasets that were generated to assess the compliance of the management of blood products to guideline 4 were left out of the analysis because of missing data. The time stamps that were generated by the tags concerning these blood products were not registered during departure or arrival of the blood products at the BTL, which resulted into incomplete datasets. Due to the missing data, the exact time stamp of the transfusion of these 12 blood products could not be calculated. The reason for this was that the tags attached to these products were not dropped into the stop box, and as a consequence, the times of transfusion required for calculating compliance of these 12 blood products to guideline 4 were not registered.

In addition, from the start of this study, the datasets generated by the RFID tags attached to RBCs that were transfused at the ICU were left out. The reason that led to the exclusion of these datasets in this field study had to do with the fact that the “stop box” in each storage room at the ICU was placed too far from the location where the transfusion at the ICU finally took place. This resulted in mixed storage and transfusion datasets concerning transfused blood products at the ICU, which could no longer be distinguished. In future installations, the RFID reader that registers that a blood product is being transfused will not be placed inside the same room where blood products are being stored. The scanner that registers transfusion of RBCs will be placed closer to the location where the actual blood transfusion has taken place.

Although the RFID tags had proven to generate accurate temperature data in a laboratory setting, the temperature data generated in this field setting might still have been incorrect.

Although the results of the laboratory tests had shown that the tags were able to monitor the whole range of temperatures that blood products could achieve in the transfusion chain and to adjust to new temperature circumstances more quickly than the RBCs themselves, we did not assess how accurate the tags adjusted to changing temperature circumstances when attached to RBCs. In the field setting, tags may have failed registration of temperatures of those blood products that at a certain point in time exceeded a temperature of 10°C, but had returned to a temperature lower than 10°C within the time frame the tag needed to adjust its own temperature. This may have resulted in an underestimation of violations concerning guideline 3. However, the speed with which tags adjust is faster than the speed with which blood products adapt their temperature to changing environment temperatures [32]. An overestimation of cases violating guideline 3 might yet have resulted from the high-temperature data, often above 10°C, generated by the tags shortly after they had been activated and allocated to a blood product at the BTL. These cases were, however, excluded from the analyses. Finally, the extent with which guideline 2 concerning the mandatory preservation of blood products in official cooling systems was violated may, in fact, have been much lower than estimated. In theory, a blood product taken from an official cooling system should be around the operating room only a few minutes before it is transfused or transported along the patient to the ICU. In practice, blood products are often stored in a nonvalidated cooling system inside the preparation room of the operating room where the patient is operated. These blood products, although unofficially, might have been preserved at recommended temperatures and subsequently transfused within 1 hour after leaving a nonofficial cooling system. This issue has been tackled by replacing the nonofficial cooling systems with official cooling systems.

Internal and external validity dimensions that are important to discuss in the translation of the results of this study to other settings are the representativeness of the particular study setting (organization of blood transfusion chain within intrahospital blood bank and operating room complex of an academic hospital), of (the behavior of) the subjects (BTL personnel, operating room personnel, and cardiothoracic patients), and of the type of RFID technology used. First, academic hospital management teams may promote a culture of evidence-based practices, optionally supported by information technologies such as RFID and perhaps more explicitly than management teams of nonacademic hospitals. If so, the negative results concerning the quality of the management of the blood transfusion chain in the academic hospital setting of the AMC of this study point out the need to conduct similar quality assessment studies in nonacademic settings. Second, we only tracked blood products for transfusion of cardiothoracic surgery patients; the volume of blood transfusions of other patient groups and consequently the organization of the blood transfusion chain might differ from that of cardiothoracic surgery patients. Blood transfusion chains may be differently organized for other reasons; not all hospitals may, for example, have BTLs available within their organization. Finally, we used a specific RFID technology to generate real-time location, time stamp, and temperature data for blood products traced in this study. The quality of data generated by other RFID technologies may differ from that of the RFID system we implemented.

To our opinion, the following measures should be taken into account in future simulated field tests on RFID avoid tag or data being lost:

- Organize human activation or deactivation activities of tags as close to the location where the corresponding activities that are to be measured take place. Or if possible, design tags that measure alterations in the process itself. That is, in our setting, the detachment of the tags from the blood bag should be registered by the tag itself instead of dropping it in the stop box through human action.
- In the real-life test “follow” the tags, by shadowing activities on site to discover other environmental factors that cannot be discovered in the simulated field test preceding the real field test. In our setting, clinicians might handle tags in another way than prescribed by procedures. On the basis of the outcomes, make adjustments accordingly and test again until the desired data quality has been reached.
- The reasons for data losses caused by human action should be discussed with the people who managed the tags more thoroughly to discover “user unfriendly” activities.

### Conclusions

In conclusion, RFID has started to make inroads into health care and is beginning to see the use to track blood for transfusions as shown in this study, to provide more extensive patient identification than traditional bar coding scan, to track and locate capital equipment within the hospital, and to track pharmaceuticals. RFID may ultimately be used for many additional functions and tremendously enhance potentials for safeguarding patient safety, continuously improving cost effectiveness and process inefficiencies by redesign efforts of work and process flows.

## Appendix

Multimedia Appendix 1 Schematic overview of red blood cells compliancy to guidelines.

## Abbreviations

AMC: Academic Medical Center
BTL: blood transfusion laboratory
ICU: intensive care unit
RBC: red blood cell
RCTs: randomized controlled trials
RFID: radio frequency identification
