# Supplementary figures and images for: The Value of Radio Frequency Identification in Quality Management of the Blood Transfusion Chain in an Academic Hospital Setting
Source: JMIR Med Inform. 2019 Aug 5;7(3):e9510. doi: 10.2196/medinform.9510 (PMC6699112; doi:10.2196/medinform.9510)

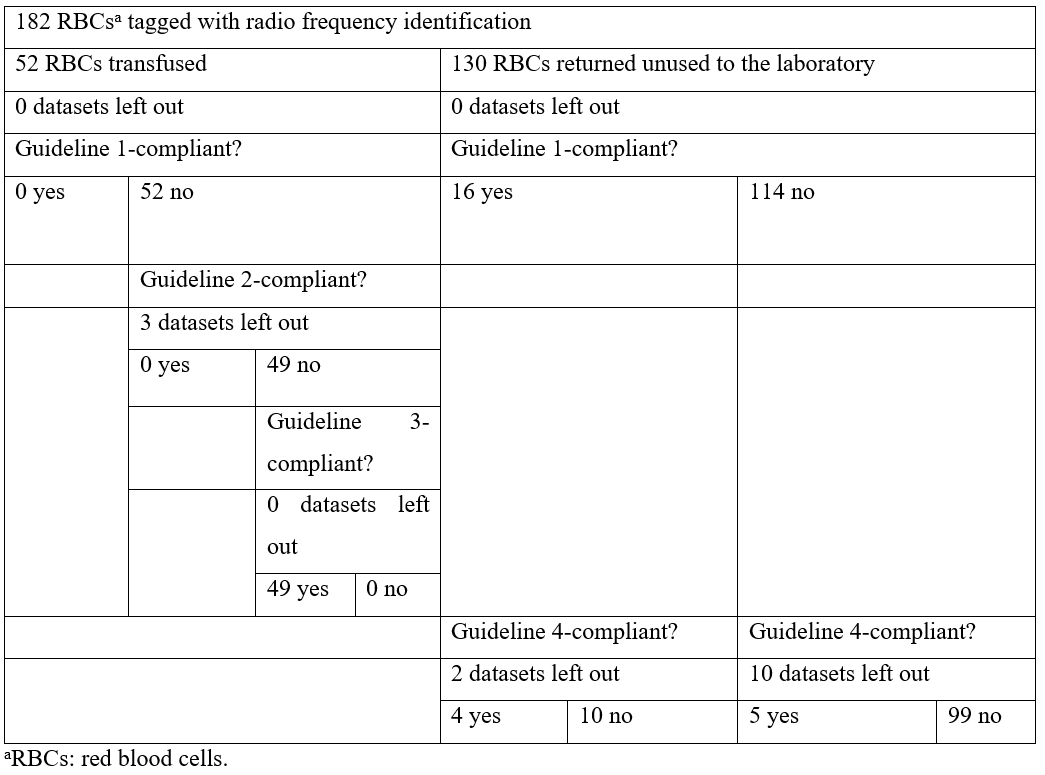

Supplement: Multimedia Appendix 1 [file medinform_v7i3e9510_app1.png]
